# Hypothalamic Hemangioma-like Pilocytic Astrocytoma in an Adult Patient: A Systematic Review with a Focus on Differential Diagnosis and Neurological Presentation

**DOI:** 10.3390/jcm13123536

**Published:** 2024-06-17

**Authors:** Roberta Costanzo, Vittoria Rosetti, Alessia Tomassini, Dalila Fuschillo, Giorgio Lofrese, Domenico Gerardo Iacopino, Luigino Tosatto, Marcello D’Andrea

**Affiliations:** 1 Neurosurgical Clinic, AOUP “Paolo Giaccone”, Post Graduate Residency Program in Neurology Surgery, Department of Biomedicine Neurosciences and Advanced Diagnostics, School of Medicine, University of Palermo, 90127 Palermo, Italy; gerardo.iacopino@gmail.com; 2 Department of Neurosurgery, Institute of Neurological Sciences of Bologna, 40124 Bologna, Italy; vittoria.rosetti@studio.unibo.it; 3 Department of Neurosurgery, M. Bufalini Hospital, 47521 Cesena, Italy; alessia.tomassini@auslromagna.it (A.T.); dalila.fuschillo@auslromagna.it (D.F.); giorgio.lofrese@auslromagna.it (G.L.); luigino.tosatto@auslromagna.it (L.T.); marcello.dandrea@auslromagna.it (M.D.)

**Keywords:** pilocytic astrocytoma, brain tumors, diagnostic challenges, glioblastoma, adults, MRI

## Abstract

**Background:** Pilocytic astrocytoma (PCA) are commonly observed as slow-growing noncancerous brain tumors in pediatric populations, but they can also occur in adults, albeit rarely. When located in diencephalic regions, particularly in the hypothalamus, they present unique diagnostic and management challenges due to their rarity and overlapping clinical and radiological features with other intracranial pathologies. This systematic review aims to provide a comprehensive understanding of hypothalamic PCA in adults, focusing on their differential diagnosis, neurological presentation, diagnostic modalities, treatment strategies. A case illustration is also described in order to better underline all the difficulties related to the diagnostic process. **Material and methods:** A systematic literature search was conducted in the PubMed/MEDLINE, Embase, and Scopus databases up to November 2023 to identify studies. **Results:** The systematic literature search identified a total of 214 articles. Following screening by title and abstract and full-text review, 12 studies were deemed eligible and are included here. **Conclusions:** Adult-onset PCA in diencephalic regions pose diagnostic challenges due to their rarity and overlapping features with other intracranial lesions. Advanced imaging techniques play a crucial role in diagnosis, while surgery remains the cornerstone of treatment. Multidisciplinary collaboration is essential for the optimal management and long-term follow-up of these patients.

## 1. Introduction

Pilocytic astrocytoma (PCA) is considered as the most prevalent brain tumor in childhood [1], even though it can be found in adults, accounting for up to 17% of cases in individuals older than 30 years [2]. Among adults, PCA comprises 5% of primary brain tumors, commonly manifesting within the cerebellum [3]. Notably, optic and hypothalamic localizations are observed in 9–30% of cases and are particularly prevalent in cases related to neurofibromatosis type 1 (NF1) [4]. Clinical presentations in adults typically involve manifestations, such as mass effect, seizures, and hydrocephalus, although PCA might be discovered incidentally [5].

Pilocytic astrocytoma represents a distinct histologic and molecular subset within gliomas, characterized by well-circumscribed, slow-growth patterns, and classified according to the World Health Organization (WHO) as grade 1 tumors. Molecular alterations in adult PCA often involve dysregulation in the MAPK pathway, with the most common alteration being the fusion of BRAF. The MAPK (mitogen-activated protein kinase) pathway and the BRAF (v-Raf murine sarcoma viral oncogene homolog B) gene are strictly involved in the pathogenesis of both hypothalamic hemangioma and pilocytic astrocytoma. This signaling cascade governs pivotal cellular processes, including proliferation, differentiation, and survival. In detail, the MAPK/ERK pathway is commonly found in various cancers, such as melanoma and glioblastoma, and is related to neurogenesis (the differentiation of mesencephalon and metencephalon), memory development, and pain perception. In the context of these tumors, mutations in the BRAF gene are one of the most common, with the V600E mutation being particularly prevalent, and these lead to the sustained activation of the MAPK pathway. This aberrant activation promotes uncontrolled cell proliferation and tumor growth by circumventing normal regulatory mechanisms. Across all the molecular alterations, the BK fusion (i.e., a gene fusion between KIAA1549 and BRAF oncogenes occurs due to 7q34 duplication) is the most common molecular abnormality in sporadic infratentorial PA affecting pediatric patients. Moreover, it is associated with a better prognosis with an optimal progression-free survival [6].

While the overall survival (OS) rate is excellent among pediatric patients, the survival rate diminishes with increasing age at diagnosis, with adults over 40 years exhibiting a 10-year OS closer to 70% [7].

In adults, contrast enhancement patterns identified on MRI might mimic aggressive lesions, potentially leading to an initial misdiagnosis of a high-grade glioma or vascular malformation [8,9]. The misdiagnosis of hypothalamic hemangioma-like pilocytic astrocytoma in adult patients might have profound implications across multiple domains of clinical care. Firstly, it may lead to inappropriate treatment modalities, potentially subjecting patients to unnecessary risks, multiple surgeries, or the delaying of effective therapies. This can directly impact treatment outcomes and patient prognosis, as the delay in administering proper treatment may allow the tumor to progress unchecked, leading to worsened outcomes and higher infection risks, due to a longer length of hospital stay, compromising quality of life. Moreover, inaccurate diagnosis can significantly affect prognostic assessments, potentially resulting in incorrect expectations about disease progression and outcomes. This misinformation can influence treatment decisions and care planning, ultimately impacting patient management and healthcare resource allocation. 

Indeed, a comprehensive knowledge of the potential clinical and radiologic characteristics in distinguishing such similar tumors is crucial for appropriate management. Thus, the authors present a systematic review focusing on the differential diagnosis and neurological presentation of hypothalamic PCA in adults, supplemented by a case illustration.

## 2. Materials and Methods

### 2.1. Literature Search Strategy

A systematic literature search was conducted using electronic databases including PubMed/MEDLINE, Embase, and Scopus to identify studies on hypothalamic pilocytic astrocytoma in adults. The search was performed using the following Medical Subject Headings (MeSH) terms: “hypothalamic pilocytic astrocytoma” AND “adults”; “hypothalamic” AND “astrocytoma”; “adults” AND “astrocytoma”. The search strategy was limited to studies published up to November 2023.

### 2.2. Study Selection Criteria

Studies were included if they met the following criteria:

- Articles published in the English language.
- Investigated hypothalamic pilocytic astrocytoma in adult patients.
- Provided detailed information on diagnostic methods, differential diagnosis, clinical presentation, treatment strategies, and outcomes.
- Included original research articles, case reports, case series, or review articles.

Studies were excluded if they met any of the following criteria:

- Not written in the English language.
- Did not specifically focus on hypothalamic pilocytic astrocytoma in adult patients.
- Lack of detailed information on diagnostic methods, differential diagnosis, clinical presentation, treatment strategies, or outcomes.
- Animal studies, editorials, letters to the editor, commentaries, conference abstracts, or reviews without original data.
- Duplicate publications or overlapping data from the same study population.
- Studies with insufficient data or inadequate reporting that precluded any meaningful extraction of relevant information.
- Studies conducted exclusively in pediatric populations or patients with other types of brain tumors not directly related to hypothalamic pilocytic astrocytoma in adults.

### 2.3. Data Extraction

Data extraction was conducted independently by two reviewers using a standardized data extraction form. Extracted data included study characteristics (e.g., author, publication year, study design), patient demographics, clinical presentation, diagnostic methods, treatment modalities, outcomes and, whenever reported, follow-up. Any discrepancies were solved by a third author.

### 2.4. Quality Assessment

The quality of included studies was assessed using appropriate tools depending on the study design. For observational studies, the Newcastle–Ottawa Scale (NOS) [10] was used, while the Joanna Briggs Institute (JBI) Critical Appraisal Checklist [11] was utilized for case reports and case series.

### 2.5. Data Synthesis

A narrative synthesis approach was employed to summarize the findings of the included studies [12]. Key themes related to diagnostic methods, clinical presentation, treatment strategies, and outcomes were identified and synthesized. Due to their heterogeneous clinical and radiological presentation, all the results were tabulated to provide a comprehensive overview of the literature on hypothalamic pilocytic astrocytoma in adults, with the aim of better focusing their management.

## 3. Results

The systematic literature search identified a total of 214 articles. Duplicated papers were removed using Microsoft Excel 16.37 Software (Redmond, WA, USA). Then, articles were screened by titles and abstracts, and non-related articles were excluded. 

**Figure 1:**
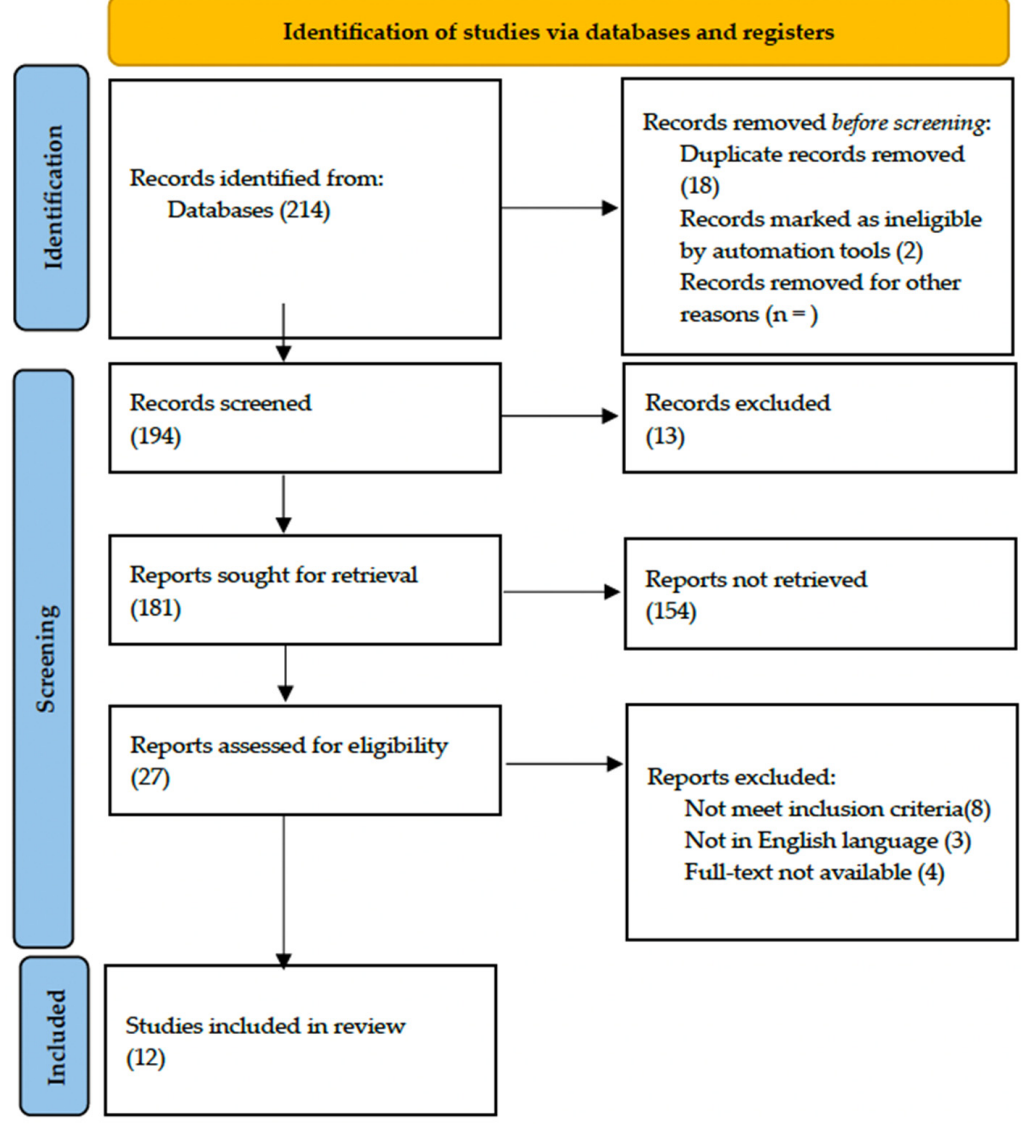
PRISMA flow diagram.

After removing duplicates and screening titles and abstracts, 27 articles were selected for full-text assessment. Following a full-text review, 8 articles were excluded because they did not meet the inclusion criteria, 3 articles were not in the English language and, in 4 articles, the full text was not available. Finally, 12 studies were deemed eligible and included in the qualitative synthesis. The Preferred Reporting Items for Systematic Reviews and Meta-Analyses (PRISMA) flow diagram detailing the study selection process is presented below (Figure 1).

### 3.1. Patients’ Demographics 

Patients’ demographics were not consistently reported across the included studies (Table 1). 

Indeed, the data set presented encompasses a varied range of patients with hypothalamic pilocytic astrocytoma, with a mean age of approximately 33.6 years (range spanning from 18 to 50 years). A total of 35% (7/20) of patients are males, while female patients represent the majority at 65% (13/20) with a male: female ratio of 0.5:1; furthermore, 1 study out of 21 (4%) did not specify gender or age. 

**Figure 2:**
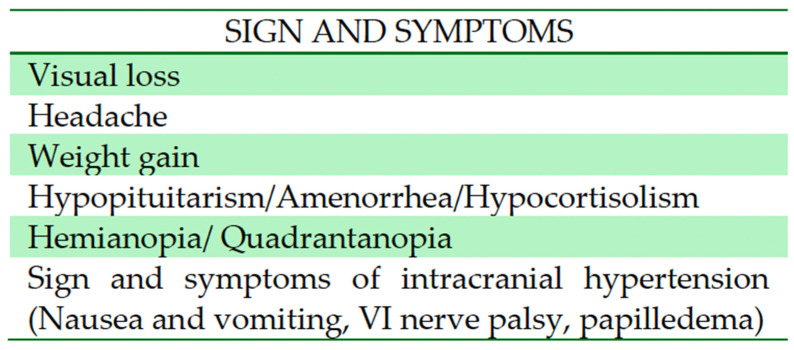
The most common symptoms found in diencephalic PCA.

**Figure 3:**
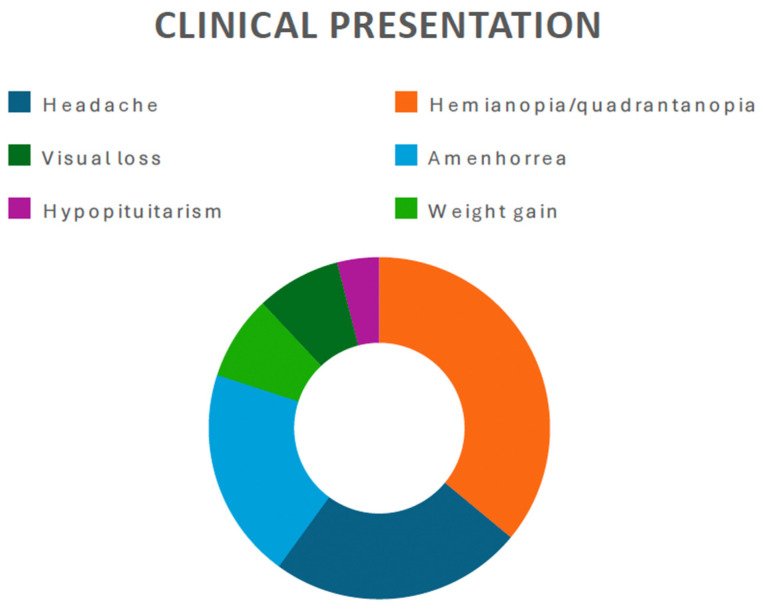
Pie chart showing the most commonly reported clinical presentation in the present literature review.

Among the various neurological presentations observed, visual impairment (i.e., hemianopia, quadrantanopia) emerges as the most prevalent, as it was reported in 52.3% of cases (11 out of 21 patients). Headache was reported in 6 patients (28.5%). Due to the relationship with the pituitary stalk, eight patients (38%) presented with endocrinological disorders (hypopituitarism, amenorrhea, or hypocortisolism) (Figure 2 and Figure 3). 

**Figure 4:**
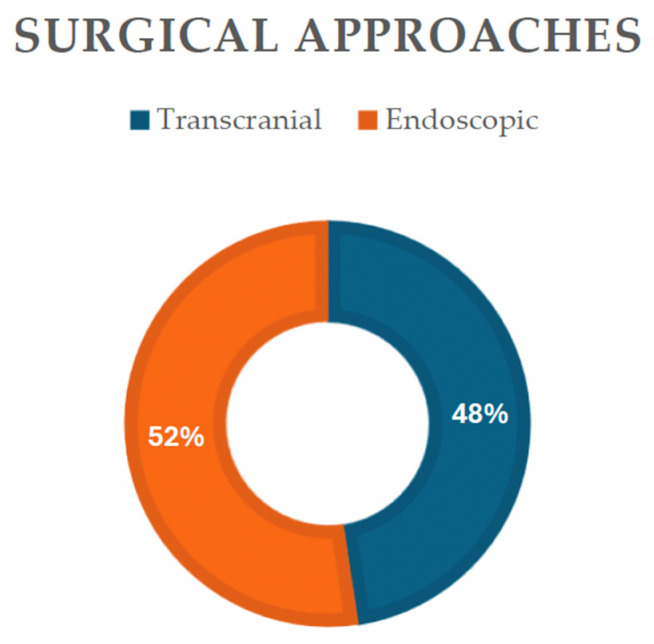
Diagram showing the surgical approaches reported in the present literature review.

Endoscopic or transcranial methods, either through a pterional or transcallosal approaches, were reported, with the endoscopic as the primary modality employed, noted in 48% of cases (11 out of 21 patients) (Figure 4), indicating their widespread use in both diagnosis and treatment. Transcranial approaches, while utilized less frequently (47%), still play a significant role in managing hypothalamic pilocytic astrocytoma.

**Figure 5:**
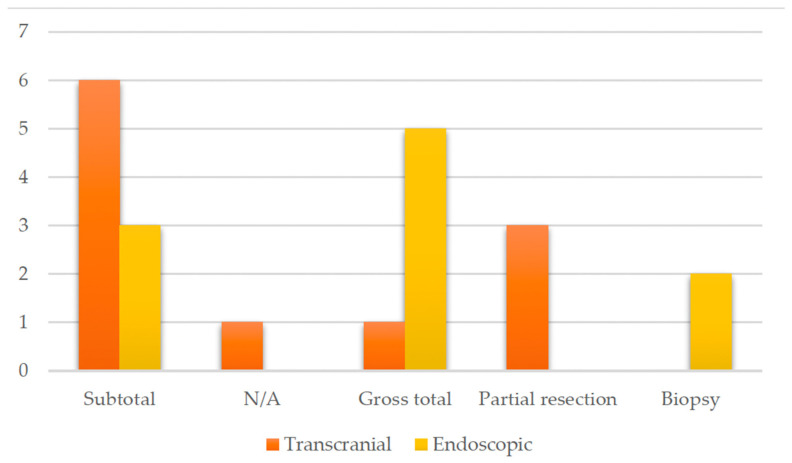
Histogram showing a comparison of the resection rate regarding the transcranial and endoscopic approaches.

Subtotal resection was performed in 49% of cases (9 out of 21 patients), followed by gross total resection (29%) and partial resection and biopsy with or without radiotherapy (Figure 5).

In the postoperative course, improved vision emerges as the most common reported outcome, and was observed in 49% of cases (9 out of 21 patients). However, complications, such as hypopituitarism, diabetes insipidus (DI), and transient DI, are reported in 38% of cases (8 out of 21 patients), highlighting the complexity of managing these lesions. Additionally, mortality attributed to hypothalamus reaction/meningitis is reported in 10% of cases (2 out of 21 studies), underscoring the critical nature of some cases.

The mean follow-up duration spans approximately 52.33 ± 68.78 months, ranging from 6 to 240 months, indicating the importance of long-term monitoring in assessing treatment efficacy and patient outcomes.

These findings provide a comprehensive overview of the demographic characteristics, clinical presentations, treatment strategies, outcomes, and follow-up data associated with hypothalamic pilocytic astrocytoma as documented in the literature.

### 3.2. Illustrative Case 

#### 3.2.1. Clinical Presentation

**Figure 6:**
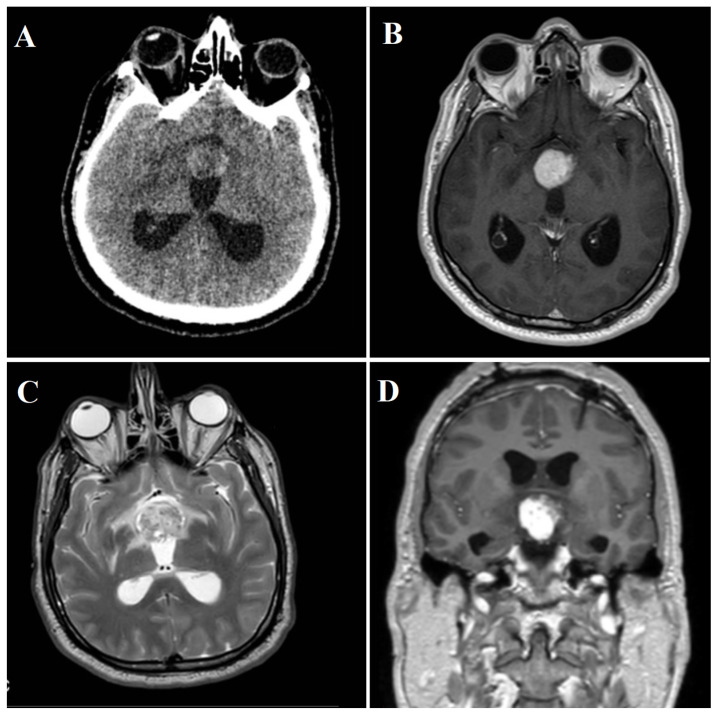
(**A**) Initial brain CT-scan showing a heterogeneous spontaneous hyperdense hypothalamic lesion with associated hydrocephalus. (**B**) Axial MRI appearance of the lesion, with intense gadolinium enhancement. (**C**) Inhomogeneous hyper-intensity on MRI T2-weighted images. (**D**) Coronal MRI appearance of the lesion, with intense gadolinium enhancement.

A 48-year-old male presented at the Emergency Department in April 2022, with an acute stuporous state and no history of neurologic disease or notable trauma. Physical examination revealed a third cranial nerve palsy on the right side. A brain computed tomography (CT) scan showed a hypothalamic lesion causing obstructive hydrocephalus (Figure 6A). CT angiogram showed no evidence of vascular abnormalities. The patient was promptly referred to the Neurosurgical Department and underwent urgent bilateral external ventricular drainage placement to firstly treat acute hydrocephalus.

#### 3.2.2. Radiological Findings

Once the patient was clinically and neurologically stable, a brain MRI was performed, revealing an expansive hypothalamic lesion infiltrating the anterior part of the third ventricle. T2-weighted images (T2WI) displayed inhomogeneous hyper-intensity, while T1-weighted images (T1WI) indicated low signal intensity. In addition to an altered signal in the optic chiasm and tracts, the lesion appeared close to the pituitary stalk. Susceptibility weighted imaging (SWI) sequences detected intralesional bleeding and microcalcification. Post-contrast administration revealed intense enhancement (Figure 6B–D), accompanied by increased perfusion indexes.

#### 3.2.3. Diagnostic Workup

In order to evaluate a hypothetical extra-cerebral origin, a whole-body CT scan was performed, disclosing sub-centimetric liver lesions and a solid-cystic mass (37 × 30 mm) in the ileocecal region. An endocrinological assessment indicated increased serum ACTH levels, initially highly supposing a metastatic neuroendocrine tumor, which was not conclusively supported upon further testing. Moreover, a CT-guided liver biopsy was performed, not yielding any diagnostic results.

#### 3.2.4. Treatment and Surgical Intervention

**Figure 7:**
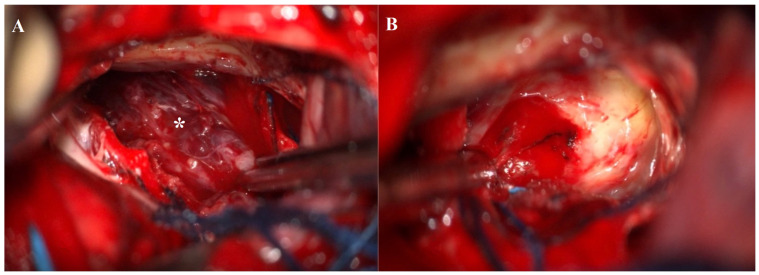
(**A**) Intraoperative image showing an encapsulated brownish, vascularized lesion (white asterisk). (**B**) Operative field after gross total resection of the lesion.

**Figure 8:**
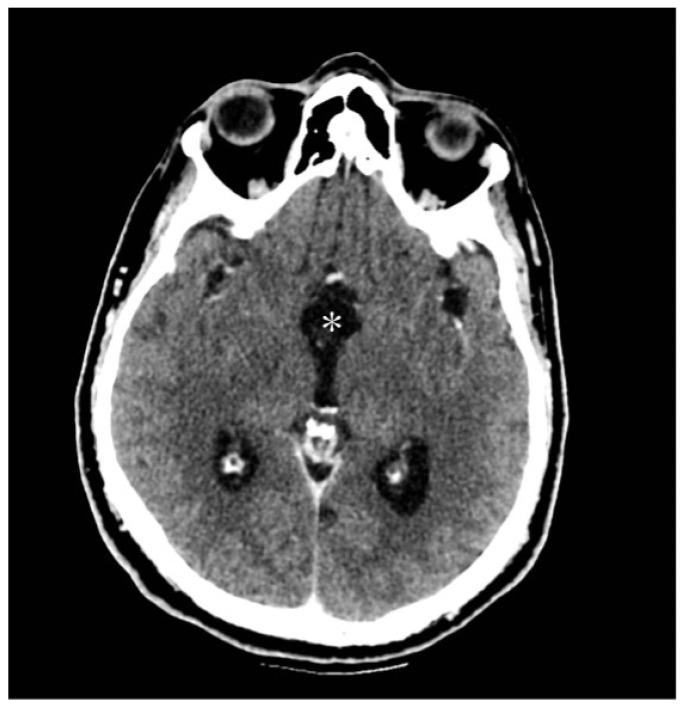
Postoperative CT brain scan showing a gross total resection (white asterisk) of the lesion with an initial decrease in ventricular size.

The patient underwent neurosurgical resection via a right parasagittal craniotomy and interhemispheric transcallosal approach. The lesion, identified as a medial protrusion beneath the septum pellucidum, was encapsulated, brownish, and vascularized. A gross-total resection was performed (Figure 7A,B) and subsequently confirmed by a postoperative CT scan (Figure 8).

Histological evaluation revealed partial necrosis and hemorrhage, with spindle cell-surrounded vessels. The diagnosis was a pilocytic astrocytoma (grade 1, according to WHO 2021) associated with a hemangioma-like vascular component, an infrequent histological feature reported in the literature. Immunostaining showed positive GFAP, Olig2, and S100, the presence of Rosenthal fibers, and a Ki-67 proliferation index of <1%.

The postoperative course was complicated by the occurrence of diabetes insipidus and hypopituitarism, necessitating hormone replacement therapy, and by a transient non-disabling memory impairment. He was discharged after one month and then addressed to a rehabilitation center.

## 4. Discussion

Pilocytic astrocytoma (PCA) are predominantly known as slow-growing brain tumors affecting pediatric populations, with an incidence rate of 0.91/100,000 per year [22]. No gender predilection has been found, with a male–female ratio 1:1. However, their manifestation in adult diencephalic regions, particularly in individuals without neurofibromatosis type 1 (NF1), is exceedingly rare and, generally, these account for 0.8% of cases in patients over 19 years [22]. They can arise from the anterior optic pathway and involve the optic chiasm, the optic radiation, and optic nerve, or arise posteriorly and involve the hypothalamus, pituitary gland, and the third ventricle. In such cases, the clinical presentation can be varied and often misleading, delaying the diagnosis. Moreover, imaging data may be misleading as well, clearly influencing the surgical approach and planning [23,24]. This article provides an in-depth exploration of the epidemiology, clinical presentation, diagnostic challenges, treatment modalities, and outcomes associated specifically with adult-onset PCA in diencephalic regions.

### 4.1. Epidemiology and Clinical Presentation

Pilocytic astrocytoma represents the most common noncancerous lesion of the pediatric population, with an incidence rate of 0.91/100,000 per year. There is not a defined gender predilection, with cerebellum, optic nerve, and chiasm as the most involved locations. Nevertheless, its occurrence is exceptionally rare in adults over 30 and even rarer in individuals over 50 years old. In children, PCAs are usually located in the infratentorial region, while in adults they are usually found in diencephalic regions, posing a unique challenge in epidemiological assessment due to their scarcity and limited reported cases. Unlike the pediatric population where PCAs are relatively common, their occurrence in adults without NF1 remains sporadic [5,25]. Clinical presentation in adults often involves nonspecific symptoms, including visual disturbances, ophthalmoplegia, hypothalamic dysfunction (such as hormonal imbalances and thermoregulatory disturbances), headaches, nausea, vomiting, and cognitive deficits. Among all the abovementioned signs and symptoms, signs of hydrocephalus might be also recognized (sixth nerve palsy, nystagmus, dysmetria, papilledema), requiring a prompt and immediate treatment when it occurs. Nonetheless, the literature data have shown that PA in adults is more aggressive, with a 5-year survival rate of 52.9% in patients over 60 years [26]. Leptomeningeal dissemination, drop metastases, or skeletal metastases were also identified, since a more aggressive behavior due to the presence of PI3K/AKT pathway alteration was observed in these patients [25]. In such cases, as shown in a Brazilian series of patients affected by PA, a longer follow-up is mandatory. They have also reported the lack of a statistically significant relationship between tumor location and its progression, which corroborates the importance of a prompt diagnosis and of an adequate extent of resection [26]. However, due to the rarity of adult-onset PCA and the overlap of symptoms with other intracranial pathologies, diagnosis can be delayed, leading to potential complications and poorer prognoses. Moreover, in addition to a delayed diagnosis, according to recent studies, other elements might influence adult patient’s outcome, such as a higher age or BMI. The latter one is statistically related, as are most extra- or intracranial tumors, to a worst prognosis due to all the factors (i.e., hyperglycemia, insulin resistance, increased level of fatty acids, and release of adipokines) that might contribute to tumoral growth and that might affect the immune system surveillance role [26,27,28].

### 4.2. Diagnostic Challenges and Imaging Modalities

Diagnosing adult-onset PCA in diencephalic regions presents significant challenges due to their nonspecific clinical presentations and overlapping radiological features with other intracranial lesions. These tumors can mimic various pathologies, including high-grade gliomas, vascular malformations, pituitary adenomas, and inflammatory or infectious processes. In cases of PCA, in fact, the optic nerve appears enlarged and elongated, with a typical “dotted I sign” with or without contrast enhancement, which should be properly distinguished from inflammatory lesions or from intracranial hypertension. Indeed, a thorough analysis of patient’s clinical history is crucial. Pain during extraocular movements, white matter abnormalities, and an increase in inflammatory indexes after visual loss followed by an immediate response to glucocorticoid may suggest an inflammatory etiology. An absence of the abovementioned factors may support the diagnosis of PCA. In cases of lymphoma, the lesion instead appears hypointense on T2-weighted images, with a restricted diffusion. Another differential diagnosis, albeit rare, is represented by optic nerve sheath meningiomas, which appears as hyperostotic lesions, which surround the nerve with the typical “tram-track sign” (i.e., the optic nerve is not enhanced while the neoplastic lesion is hyperintense) [29,30,31,32]. Therefore, a multidisciplinary approach involving neurologists, neurosurgeons, neuroradiologists, and neuropathologists is essential for accurate diagnosis and treatment planning. 

Advanced neuroimaging techniques play a critical role in the evaluation of adult-onset PCAs. Magnetic resonance imaging (MRI) with contrast enhancement remains pivotal, providing detailed anatomical information and aiding in differential diagnosis [32,33,34,35]. Characteristic MRI features of PCA may include a cystic mass with a mural nodule and well-defined margins. The presence of a hemorrhage is rare, but it may also occur, associated with variable enhancement patterns, reflecting its heterogeneous nature. Nevertheless, adults’ radiological presentation might be slightly different from the abovementioned imaging characteristics, leading to a difficult differential diagnosis just based on age and tumor location [35,36,37]. Among all the radiological tools available, the role of radiomics is assuming a deepening importance and might be helpful in the future to better understand and focus imaging data, thanks to advanced algorithms used to aid decision-making processes. Various studies have already started to experiment, building a “decision tree model” to evaluate PCA and differentiate it from glioblastoma [37]. These models can, indeed, thanks to biological information, be helpful for prognosis, diagnosis, and therapies, with high accuracy. Additionally, diffusion-weighted imaging (DWI) and perfusion MRI (MRP) serve as valuable adjuncts in distinguishing PCAs from other intracranial lesions [8,36]. Firstly, DWI assesses the random motion of water molecules within tissues, typically demonstrating higher apparent diffusion coefficient (ADC) values in PCAs compared to high-grade gliomas due to their lower cellular density and less restricted diffusion. Then, perfusion-weighted imaging of pilocytic astrocytoma has shown a lower value of relative cerebral blood volume (rCBV) (generally 1,7) in comparison to “physiologically appearing” white matter. This condition is probably related to the open tight junction of endothelial cells typically found in PCAs, confirming lower rCBV in low-grade glioma compared to other intracranial tumors. MRP provides insights into tumor vascularity and blood–brain barrier permeability, with PCA often exhibiting characteristic perfusion patterns indicative of their low-grade nature [8,37,38]. In MRI spectroscopy, PCAs show higher level of choline and lactate, probably due to a mitochondrial dysfunction which alters electrons’ behavior and oxidative phosphorylation. Creatine levels, usually related to the infiltrative nature of glial tumors, are low, leading to a decrease in N-acetyl aspartate/creatine, choline/creatine ratios in PCA. Moreover, another interesting detail is that spectroscopy metabolite profiles vary according to tumor location (supratentorial versus cerebellar location, which shows low levels of myoinositol and glutamine) [29].

### 4.3. Treatment Strategies

The endoscopic endonasal approach (EEA) has emerged as a valuable surgical technique for accessing and resecting tumors in the diencephalic region, including PCA involving the hypothalamus, particularly in adults. EEA offers several advantages, including direct access to the tumor, minimal brain manipulation, reduced risk of neurological deficits, and improved visualization of critical structures, such as the optic nerves, chiasm, and hypothalamic nuclei, without any damages and with no need to open the lamina terminalis [16,21]. Reports of successful treatment utilizing EEA in adult patients underscore its efficacy and safety in achieving tumor resection while preserving neurological function. Among these cases, Zhuo-Ya Zhou et al. [21] have described five female patients with hypothalamic PCAs treated via an endoscopic endonasal approach (EEA), obtaining a successful gross total resection in four out of five patients. Nevertheless, according to Zoli et al., not all the PCA can be treated through an EEA, since the endoscopic route is strictly subjected to the position of the chiasm. Its interposition can lead to a narrow corridor that is difficult to overcome, so it is preferable in these cases to use a transcranial approach [16]. Another important issue to face is the surgeon’s expertise, which might influence the approach chosen. A narrower corridor can interfere with a maximal resection, especially when a cleavage plane is difficult to find. Therefore, surgical intervention remains the cornerstone of treatment for adult-onset PCAs in diencephalic regions, aiming to achieve maximal safe resection while preserving neurological function. Neurosurgeons face the challenge of navigating delicate neurovascular structures to minimize the risk of neurological deficits while maximizing tumor resection. Preoperative neuroimaging and intraoperative neurophysiological monitoring play pivotal roles in surgical planning and in assessing neurological integrity during surgery. While the goal of surgery is often gross total resection (GTR), achieving this objective can be challenging due to the infiltrative nature of PCA and their proximity to critical structures. In hypothalamic or chiasmatic PCA, complete resection is often difficult due to the risk of injury of various delicate structures, such as the pituitary gland, carotid arteries, and the hypothalamus. 

In cases where complete resection carries a high risk of neurological morbidity, subtotal resection or debulking surgery may be considered to alleviate mass effect, relieve symptoms, and facilitate adjuvant therapies. Moreover, according to this systematic review, the higher rate of GTR was obtained through an endonasal route, underlying the need for a better understanding of and thorough investigations into the behavior of PCA in adults’ patients.

In addition to the chance to obtain a subtotal resection, according to Bartels et al., a high microvascular density might be evaluated and associated to recurrence, requiring a more careful treatment and closer follow-up [39].

Following surgery, close postoperative monitoring is essential to assess neurological function, manage potential complications, and guide adjuvant treatment strategies in adult patients. Adjuvant therapies, including radiotherapy and chemotherapy, may be recommended based on certain factors, such as residual tumor burden, tumor location, histological features, and patient-specific risk factors [26]. Indeed, the role of chemotherapy is to avoid further endocrinological or visual impairment by decreasing the size of the tumor. The most commonly used chemotherapy drugs are carboplatin, vincristine, and trametinib, with excellent results. The use of radiotherapy is still controversial due to the side effects reported, related to the procedure itself (hormonal dysfunction, cognitive impairment, and the development of other tumors, such as meningiomas or glioblastomas) [26].

Despite advances in surgical techniques and perioperative care, the management of adult-onset PCA remains challenging, and outcomes can vary widely depending on tumor characteristics, the extent of resection, and patient-related factors. Long-term follow-up care is crucial for monitoring disease progression, managing treatment-related sequelae, and addressing the evolving needs of adult patients with PCA. Multidisciplinary care teams play a pivotal role in providing comprehensive support and optimizing outcomes for these patients.

## 5. Limitations

The number of cases reviewed and analyzed in this study is limited, due to the rare occurrence of these tumors in adult patients. Indeed, such a small sample may not provide a comprehensive representation of the disease’s clinical variability and treatment outcomes. Due to the rarity of the condition, treatment strategies may vary significantly between institutions and individual cases. The study may not incorporate comprehensive genetic and molecular analyses, which are increasingly important for understanding the pathophysiology and potential targeted therapies for PCAs. Studies are selected between 1994 and 2021, determining differences in imaging modalities, techniques, and interpretations among institutions and over time, thus influencing the variability in diagnostic accuracy. The outcomes of surgical interventions, particularly those involving advanced techniques, like the endoscopic endonasal approach, are highly dependent on the surgeon’s expertise and experience. Variability in surgical skill and experience across different centers can impact the generalizability of the findings. 

## 6. Future Directions

Advancements in managing adult-onset hypothalamic PCAs rely on multifaceted avenues of inquiry and clinical innovation. Firstly, exploring the molecular landscape of adult-onset PCAs, with a focus on genetic aberrations, like BRAF fusion and dysregulation within the MAPK pathway, is crucial. Indeed, a thorough evaluation of DNA methylomic and transcriptomic analyses along with histopathological evaluation may represent a turning point in research into all the prognostic variables that might influence tumor progression [40]. These insights could lead to precision-targeted therapies, reshaping treatment strategies and potentially reducing the choice of aggressive surgeries. Concurrently, when assessing all the neuroimaging techniques, including radiomics and advanced MRI sequences, we see that the diagnostic accuracy and differential diagnosis of adult-onset PCAs is rapidly evolving. By using these methods to identify more precise imaging biomarkers, clinicians can enhance early detection, treatment planning, and disease monitoring [34,35,41]. Moreover, optimizing surgical techniques, especially in endoscopic endonasal approaches and minimally invasive procedures, is essential. These efforts aim to improve the safety and effectiveness of tumor removal while reducing postoperative neurological complications. Collaboration among neurosurgical, neuroradiological, and neuro-oncological specialties is critical for advancing surgical outcomes and preserving patients’ neurological function. Additionally, a comprehensive exploration of adjuvant therapies, like chemotherapy and radiotherapy, is pivotal. Understanding their impact on tumor control, survival, and treatment-related side effects is essential for tailoring treatment approaches based on individual tumor characteristics and patient needs. Lastly, ongoing longitudinal follow-up studies are crucial for assessing treatment effectiveness, monitoring long-term effects, and identifying predictors of disease progression [26,27]. Through rigorous longitudinal assessments, researchers can gain insights into the dynamic nature of adult-onset PCAs and refine treatment strategies accordingly. In summary, advancing the management of adult-onset hypothalamic PCAs requires a coordinated effort across scientific and clinical domains to guarantee an optimal patient outcome.

## 7. Conclusions

In conclusion, pilocytic astrocytoma of the hypothalamic/optic–chiasmatic region, without neurofibromatosis type 1 (NF1) represents a unique subset of brain tumors in adults. While the existing literature covers various aspects of these tumors, including their clinical presentation and treatment outcomes, there is a notable gap in the emphasis on the importance and description of differential diagnosis. Misdiagnosis can lead to delayed treatment, influencing the decision-making processes. Therefore, advanced imaging techniques, such as functional MRI and radiomics, along with comprehensive diagnostic models, including radiological and histopathological analyses, are indispensable for the precise diagnosis and differentiation of PCA from its mimickers. A thorough understanding of the radiological features and their correlation with clinical findings is imperative for optimal management planning. Given the potential for favorable outcomes with GTR, early and accurate diagnosis through sophisticated imaging modalities and comprehensive assessment strategies is paramount. Therefore, future research should consider a multidisciplinary approach involving neuroradiologists, neurosurgeons, and neuropathologists, as these roles are essential for navigating the diagnostic intricacies and ensuring the optimal management of adult PCA cases.

## Figures and Tables

**Table 1:** Literature data about patients showing hypothalamic PCA. DI, diabetes insipidus; N/A, not available; CSF, cerebrospinal fluid.

Author and Year	Study Design	Age	Sex	Neurological Presentation	Treatment	Diagnostic Method	Outcome	Follow Up	Techique
Valdueza et al., 1994 [13]	Original article	N/A	N/A	N/A	N/A	T1-weighted images show low-intensity masses with marked enhancement after administration of gadolinium	N/A	4 year	Transcranial
Matsumoto et al., 1997 [14]	Case report	45	Male	Headache, bitemporal hemianopia	Subtotal	T1-isointense mass, high intensity center suggesting subacute hemorrhage, irregular contrast enhancement	Improved vision	6 months (residual tumor stable)	Transcranial
Bakhoyor et al., 2000 [15]	Case report	19	Female	Headache, dizziness, lightheadedness	Partial resection	Densely and uniformly enhancing lesion in the right frontal horn of the lateral ventricle	None	6 months (involution of the tumor)	Transcranial
Bakhoyor et al., 2000 [15]	Case report	21	Female	Right homonymous hemianopia	Partial resection	Heterogenous enhancing mass above the optic chiasm in the region of the hypothalamus	Improvement in visual deficit	6 months (involution of the tumor)	Transcranial
De Divitiis et al., 2007 [16]	Original article Case series	43	Female	N/A	Subtotal	N/A	None	N/A	Endoscopic
Arbolay et al., 2009 [17]	Original article, case series	42	Male	Headache	Biopsy	N/A	Died due to meningitis	N/A	Endoscopic
Paluzzi et al., 2011 [18]	Technical note	44	Male	Headache, amenhorrea	Subtotal	Pituitary lesion attached to the pituitary stalk	Adrenal insufficiency and DI	N/A	Endoscopic
Zoli et al., 2014 [19]	Original article, case series	38	Male	Progressive visual loss, hypopituitarism	Gross total	Mixed and solid cystic mass extending within the ventricle and displacing the optic chiasm	CSF leak, DI, improvement in visual deficit	17 months	Endoscopic
Zoli et al., 2014 [19]	Original article, case series	42	Male	Homonymous hemianopia	Subtotal	N/A	Improved vision, hypopituitarism, and DI	81 months	Endoscopic
Zoli et al., 2014 [19]	Original article, case series	23	Female	Bitemporal hemianopia and acuity deficit	Biopsy+ Radiotherapy	Highly suggestive of high-grade glioma	Vision normalized, DI	45 months	Endoscopic
Abou Al-Shaar et al., 2016 [20]	Case report	30	Male	Hypocortisolism, visual defect,	Partial Resection	Sellar– suprasellar solid and cystic lesions which displace the infundibulum	None	6 months	Transcranial
Hidalgo et al., 2018 [21]	Case report	50	Female	Personality change, weight gain, incomplete right homonymous hemianopia	Gross total	Enhancing lesion along the optic tract with a small cystic component	Transient DI, improvement in visual field	20 year	Transcranial
Bin Abdulquader et al., 2018 [22]	Original article, case series	32	Female	Hallucinations, nausea and vomiting	Subtotal	N/A	Transient DI, improvement in visual field	7 months	Endoscopic
Shoji et al., 2020 [23]	Original article, case series	18	Male	Right hemianopia	Subtotal	N/A	None	11 year	Transcranial
Shoji et al., 2020 [23]	Original article, case series	19	Female	Right upper quadrantanopia	Subtotal	N/A	None	10 year	Transcranial
Shoji et al., 2020 [23]	Original article, case series	26	Female	Right lower quadrantanopia	Subtotal	Enhanced lesion in the optic chiasma extending to the third ventricle	None	14 year	Transcranial
Shoji et al., 2020 [23]	Original article, case series	36	Female	None	Subtotal	N/A	None	1 year	Transcranial
Zhou et al., 2021 [24]	Original article, case series	20	Female	Headache, amenhorrea, weight gain	Gross total	Suprasellar tumor with solid and cystic portions	Hyperprolactinemia, improved vision	1 year	Endoscopic
Zhou et al., 2021 [24]	Original article, case series	41	Female	Amenhorrea, dizziness, memory deterioration	Gross total	Enhanced suprasellar, interpeduncular, and prepontine cistern lesion	Hypopituitarism, improved vision	1 year	Endoscopic
Zhou et al., 2021 [24]	Original article, case series	22	Female	Menstrual disorder	Gross total	N/A	Hypopituitarism, no change in vision	1 year	Endoscopic
Zhou et al., 2021 [24]	Original article, case series	46	Female	Bilateral visual disturbance, headache, amenhorrea	Gross total	Giant hypothalamic tumor with no clear margin between the tumor and hypothalamic structure	Dead (due to hypothalamus reaction)	1 year	Endoscopic
Present case	/	48	Male	Third nerve palsy, stuporous state	Gross total	SWI_sequences with intralesional bleeding, microcalcification. Post-contrast administration revealed intense enhancement and increased perfusion indexes	Transient DI, hypopituitarism	1 year	Transcranial

## Data Availability

No new data were created.

## References

[1] Ostrom Q.T., Gittleman H., Liao P., Rouse C., Chen Y., Dowling J., Wolinsky Y., Kruchko C., Barnholtz-Sloan J. (2014). CBTRUS statistical report: Primary brain and central nervous system tumors diagnosed in the United States in 2007–2011. Neuro Oncol..

[2] Bell D., Chitnavis B.P., Al-Sarraj S., Connor S., Sharr M.M., Gullan R.W. (2004). Pilocytic astrocytoma of the adult–clinical features, radiological features, and management. Br. J. Neurosurg..

[3] Knight J., De Jesus O. (2024). Pilocytic Astrocytoma. StatPearls [Internet].

[4] Collins V.P., Jones D.T., Giannini C. (2015). Pilocytic astrocytoma: Pathology, molecular mechanisms, and markers. Acta Neuropathol..

[5] Bond K.M., Hughes J.D., Porter A.L., Orina J., Fang S., Parney I.F. (2018). Adult Pilocytic Astrocytoma: An Institutional Series and Systematic Literature Review for Extent of Resection and Recurrence. World Neurosurg..

[6] Gregory T.A., Chumbley L.B., Henson J.W., Wheeler B.J. (2021). Adult pilocytic astrocytoma in the molecular era: A comprehensive review. CNS Oncol..

[7] Bornhorst M., Frappaz D., Packer R.J. (2016). Pilocytic astrocytomas. Handbook of Clinical Neurology.

[8] Kikuchi K., Hiwatashi A., Togao O., Yamashita K., Kamei R., Kitajima M., Kanoto M., Takahashi H., Uchiyama Y., Harada M. (2018). Usefulness of perfusion- and diffusion-weighted imaging to differentiate between pilocytic astrocytomas and high-grade gliomas: A multicenter study in Japan. Neuroradiology.

[9] Fulham M.J., Melisi J.W., Nishimiya J., Dwyer A.J., Di Chiro G. (1993). Neuroimaging of juvenile pilocytic astrocytomas: An enigma. Radiology.

[10] Stang A. (2010). Critical evaluation of the Newcastle-Ottawa scale for the assessment of the quality of nonrandomized studies in meta-analyses. Eur. J. Epidemiol..

[11] Munn Z., Moola S., Lisy K., Riitano D., Tufanaru C. (2015). Methodological guidance for systematic reviews of observational epidemiological studies reporting prevalence and incidence data. Int. J. Evid. Based Healthc..

[12] Snilstveit B., Oliver S., Vojtkova M. (2012). Narrative approaches to systematic review and synthesis of evidence for international development policy and practice. J. Dev. Eff..

[13] Valdueza J.M., Lohmann F., Dammann O., Hagel C., Eckert B., Freckmann N. (1994). Analysis of 20 primarily surgically treated chiasmatic/hypothalamic pilocytic astrocytomas. Acta Neurochir..

[14] Matsumoto K., Akagi K., Abekura M., Maeda Y., Kitagawa M., Ryujin H., Iwasa N. (1997). Hypothalamic Pilocytic Astrocytoma Presenting with Intratumoral and Subarachnoid Hemorrhage. Neurol. Med.-Chir..

[15] Balkhoyor K.B., Bernstein M. (2000). Involution of diencephalic pilocytic astrocytoma after partial resection. Report of two cases in adults. J. Neurosurg..

[16] de Divitiis E., Cavallo L.M., Cappabianca P., Esposito F. (2007). Extended endoscopic endonasal transsphenoidal approach for the removal of suprasellar tumors: Part 2. Neurosurgery.

[17] Arbolay O.L., González J.G., González R.H., Gálvez Y.H. (2009). Extended Endoscopic Endonasal Approach to the Skull Base. MIN-Minim. Invasive Neurosurg..

[18] Paluzzi A., Fernandez-Miranda J.C., Pinheiro-Neto C., Alcocer-Barradas V., Lopez-Alvarez B., Gardner P., Snyderman C. (2011). Endoscopic Endonasal Infrasellar Approach to the Sellar and Suprasellar Regions: Technical Note. Skull Base.

[19] Zoli M., Mazzatenta D., Valluzzi A., Marucci G., Acciarri N., Pasquini E., Frank G. (2014). Expanding indications for the extended endoscopic endonasal approach to hypothalamic gliomas: Preliminary report. Neurosurg. Focus.

[20] Al-Shaar H.A., Raheja A., Palmer C.A., Schmidt M.H., Couldwell W.T. (2016). Hypothalamic–Optochiasmatic Pilocytic Astrocytoma Associated with Occipital and Sacral Spinal Cavernomas: A Mere Coincidence or a True Association?. World Neurosurg..

[21] Hidalgo E.T., McQuinn M.W., Wisoff J.H. (2018). Regression after subtotal resection of an optic pathway glioma in an adult without adjuvant therapy: Case report. J. Neurosurg..

[22] Bin Abdulqader S., Al-Ajlan Z., Albakr A., Issawi W., Al-Bar M., Recinos P.F., Alsaleh S., Ajlan A. (2019). Endoscopic transnasal resection of optic pathway pilocytic astrocytoma. Child’s Nerv. Syst. ChNS Off. J. Int. Soc. Pediatr. Neurosurg..

[23] Shoji T., Kanamori M., Saito R., Watanabe Y., Watanabe M., Fujimura M., Ogawa Y., Sonoda Y., Kumabe T., Kure S. (2020). Frequent Clinical and Radiological Progression of Optic Pathway/Hypothalamic Pilocytic Astrocytoma in Adolescents and Young Adults. Neurol. Med.-Chir..

[24] Zhou Z.-Y., Wang X.-S., Gong Y., Musyafar O.L.A., Yu J.-J., Huo G., Mou J.-M., Yang G. (2021). Treatment with endoscopic transnasal resection of hypothalamic pilocytic astrocytomas: A single-center experience. BMC Surg..

[25] Mair M.J., Wöhrer A., Furtner J., Simonovska A., Kiesel B., Oberndorfer S., Ungersböck K., Marosi C., Sahm F., Hainfellner J.A. (2020). Clinical characteristics and prognostic factors of adult patients with pilocytic astrocytoma. J. Neuro-Oncol..

[26] Salles D., Laviola G., Malinverni A.C.d.M., Stávale J.N. (2020). Pilocytic Astrocytoma: A Review of General, Clinical, and Molecular Characteristics. J. Child Neurol..

[27] Matyja E., Grajkowska W., Stępień K., Naganska E. (2016). Heterogeneity of histopathological presentation of pilocytic astrocytoma–diagnostic pitfalls. A review. Folia Neuropathol..

[28] Nair P., Murali S.H., Venkat E.H., Poyuran R. (2023). Endoscopic Endonasal Transcavernous Posterior Clinoidectomy with Interdural Pituitary Transposition for a Suprasellar Optic Pathway Pilocytic Astrocytoma: 2-Dimensional Operative Video. Oper. Neurosurg..

[29] Li X., Moreira D.C., Bag A.K., Qaddoumi I., Acharya S., Chiang J. (2023). The clinical and molecular characteristics of progressive hypothalamic/optic pathway pilocytic astrocytoma. Neuro-Oncology.

[30] Gaha M., Bouzayen F., Limam Y., Mokni M., Jemni-Gharbi H., Tlili-Graiess K. (2017). Pilocytic astrocytoma mimicking cavernous angioma: Imaging features and histological characteristics. Neurochirurgie.

[31] Hong C.S., Lehman N.L., Sauvageau E. (2014). A Pilocytic Astrocytoma Mimicking a Clinoidal Meningioma. Case Rep. Radiol..

[32] Skipworth J.R., Hill C.S., Jones T., Foster J., Chopra I., Powell M. (2012). Pilocytic astrocytoma mimicking craniopharyngioma: A case series. Ann. R. Coll. Surg. Engl..

[33] Dutta G., Singh D., Singh H., Sachdeva D., Kumar V., Chaturvedi A. (2020). Pilocytic astrocytoma of the cerebellopontine angle mimicking vestibular schwannoma: Report of a rare entity. Br. J. Neurosurg..

[34] Gaudino S., Martucci M., Russo R., Visconti E., Gangemi E., D’Argento F., Verdolotti T., Lauriola L., Colosimo C. (2017). MR imaging of pilocytic brain astrocytoma: Beyond the stereotype of benign astrocytoma. Child’s Nerv. Syst..

[35] Kumar A.J., Leeds N.E., Kumar V.A., Fuller G.N., Lang F.F., Milas Z., Weinberg J.S., Ater J.L., Sawaya R. (2010). Magnetic Resonance Imaging Features of Pilocytic Astrocytoma of the Brain Mimicking High-Grade Gliomas. J. Comput. Assist. Tomogr..

[36] Nakano Y., Yamamoto J., Takahashi M., Soejima Y., Akiba D., Kitagawa T., Ueta K., Miyaoka R., Umemura T., Nishizawa S. (2015). Pilocytic astrocytoma presenting with atypical features on magnetic resonance imaging. J. Neuroradiol..

[37] Dong F., Li Q., Xu D., Xiu W., Zeng Q., Zhu X., Xu F., Jiang B., Zhang M. (2019). Differentiation between pilocytic astrocytoma and glioblastoma: A decision tree model using contrast-enhanced magnetic resonance imaging-derived quantitative radiomic features. Eur. Radiol..

[38] Vats N., Sengupta A., Gupta R.K., Patir R., Vaishya S., Ahlawat S., Saini J., Agarwal S., Singh A. (2023). Differentiation of Pilocytic Astrocytoma from Glioblastoma using a Machine-Learning framework based upon quantitative T1 perfusion MRI. Magn. Reson. Imaging.

[39] Bartels U., Hawkins C., Ma J., Ho M., Dirks P., Rutka J., Stephens D., Bouffet E. (2006). Vascularity and angiogenesis as predictors of growth in optic pathway/hypothalamic gliomas. J. Neurosurg. Pediatr..

[40] Liu H., Chen Y., Qin X., Jin Z., Jiang Y., Wang Y. (2022). Epidemiology and Survival of Patients with Optic Pathway Gliomas: A Population-Based Analysis. Front. Oncol..

[41] Park Y.W., Kim D., Eom J., Ahn S.S., Moon J.H., Kim E.H., Kang S.G., Chang J.H., Kim S.H., Lee S.K. (2021). A diagnostic tree for differentiation of adult pilocytic astrocytomas from high-grade gliomas. Eur. J. Radiol..

